# MRI Guided Focused Ultrasound-Mediated Delivery of Therapeutic Cells to the Brain: A Review of the State-of-the-Art Methodology and Future Applications

**DOI:** 10.3389/fneur.2021.669449

**Published:** 2021-06-17

**Authors:** Nabid Ahmed, Dheeraj Gandhi, Elias R. Melhem, Victor Frenkel

**Affiliations:** Department of Diagnostic Radiology and Nuclear Medicine, and Department of Neuroradiology, University of Maryland School of Medicine, Baltimore, MD, United States

**Keywords:** central nervous system diseases, cellular therapy, MRI-guided focused ultrasound, blood-brain barrier, cellular tracking

## Abstract

Stem cell and immune cell therapies are being investigated as a potential therapeutic modality for CNS disorders, performing functions such as targeted drug or growth factor delivery, tumor cell destruction, or inflammatory regulation. Despite promising preclinical studies, delivery routes for maximizing cell engraftment, such as stereotactic or intrathecal injection, are invasive and carry risks of hemorrhage and infection. Recent developments in MRI-guided focused ultrasound (MRgFUS) technology have significant implications for treating focal CNS pathologies including neurodegenerative, vascular and malignant processes. MRgFUS is currently employed in the clinic for treating essential tremor and Parkinson's Disease by producing precise, incisionless, transcranial lesions. This non-invasive technology can also be modified for non-destructive applications to safely and transiently open the blood-brain barrier (BBB) to deliver a range of therapeutics, including cells. This review is meant to familiarize the neuro-interventionalist with this topic and discusses the use of MRgFUS for facilitating cellular delivery to the brain. A detailed and comprehensive description is provided on routes of cell administration, imaging strategies for targeting and tracking cellular delivery and engraftment, biophysical mechanisms of BBB enhanced permeability, supportive proof-of-concept studies, and potential for clinical translation.

## Introduction

### Neurologic Cellular Therapies

Treating CNS disorders with cells were trialed first in the late 1980s, when patients with Parkinson's disease (PD) and Huntington's disease (HD) underwent intrastriatal injections of fetal mesencephalic tissue. Modest improvements in motor and cognitive function were noted in PD patients, but survival of transplanted fetal dopaminergic cells was low, and a cohort of patients also developed post-engraftment dyskinesias, possibly due to patchy reinnervation (1). Engraftment was verified via increased PET signaling in HD patients (2), but one patient was noted to have graft tissue overgrowth in a 5-year follow-up, demonstrating a potential risk of fetal tissue implantation (3). Despite these limitations, the pilot trials motivated future research into using exogenous cells to treat CNS disease, especially from sources that did not raise the ethical concerns involved with fetal tissue. Over time, the consensus on the mechanistic goal of this strategy shifted from outright cell replacement toward the inclusion of more complex functions, including local immunomodulation, inducing differentiation of endogenous stem cells, or encapsulating small molecular drugs for controlled release. Compared to other therapeutic vehicles (liposomes and nanoparticles), the biological machinery of a therapeutic cell can be exploited for their natural signaling networks, migration behaviors, and endosomal compartmentalization. Moreover, cells can be genetically engineered *in vitro* to express neurotrophic factors (4), enzymes to convert innocuous prodrugs into active forms for targeted therapies (5), or chimeric antigen ligands designed to target specific pathologic cell markers for more targeted therapy (6). They can also be designed to contain built-in suicide genes to ensure that they are not retained longer than intended or undergo mutation (7).

### Stem Cells

Research probing the biochemical and mechanical underpinnings of stem cell differentiation continues to grow with ever increasing preclinical and clinical studies. Stem cells maintain their definition as undifferentiated cells with self-renewal capacity that are mainly classified based on “potency,” or capacity to develop into one or all of the three germ layers; further classification schemes are based on sourcing technique or location. Human Embryonic Stem Cells (hESCs) and induced Pluripotent Stem Cells (iPSCs) exhibit pluripotency i.e., capacity to differentiate into somatic cells of all three embryonic germ layers. Applications employing hESCs, which are derived from the embryonic inner cell mass, are limited due to ethical constraints and the risk of tumorigenicity if not fully differentiated into the tissue of interest. iPSCs are created via transfecting somatic cells (e.g., from the skin or peripheral blood) with reprogramming transcriptional factors (8). Clinical grade, human PSCs (hPSCs) for direct differentiation into midbrain dopamine neurons, for example, are currently being developed for the treatment of PD (9). While patient-derived lines that circumvent immune rejection is promising, challenges still include maximizing reprogramming efficiency and overcoming costs of expansion and safety testing (10, 11). Adult stem cells (neural, mesenchymal, hematopoietic, colonic epithelial) exhibit multipotency i.e., capacity to differentiate into a somatic cell of their respective germ layer. These reside within “stem cell niches” that have been identified in several organ tissues and either continuously proliferate and differentiate (e.g., colonic stem cells) or lie dormant until receiving molecular cues after injury (12).

Neural stem cells (NSCs) and Mesenchymal stem cells (MSCs) are common stem cell types under study for neurologic cell therapies. NSCs were discovered to reside within the subventricular zone of the lateral ventricles and subgranular zone of the dentate gyrus, two areas of adult neurogenesis which have been implicated in learning, memory and mood regulation (13, 14). The NSC migratory and differentiation functions are influenced by a network of supportive cells that provide synaptic input, transcriptional signals, and epigenetic cues (15). NSCs have been therapeutically exploited for their cell replacement potential in becoming neuronal or glial progenitor cells, producing neurotrophic factors that promote neuronal growth, and for delivering a variety of anticancer payloads (16). Isolating large therapeutic quantities of autologous NSCs directly from a patient, however, is challenging. Two recently developed harvesting techniques are being evaluated comparatively. One involves directly transforming adult somatic cells into a NSC (i.e., “transdifferentiation”) (17), while the other differentiates iPSCs to create “induced” NSCs (iNSCs) (18). MSCs reside in multiple areas in the body including bone marrow, dental pulp, adipose tissue, umbilical cord blood and amniotic fluid, conferring their ability to be sourced relatively easier than NSCs. MSCs have been shown to cross the BBB and home to primary and metastatic tumors of the brain through chemokine signaling (19, 20). The pleiotropic functions of MSCS (growth factor secretion, immunomodulation, neuroprotection, angiogenesis, anti-apoptosis, inducing differentiation) (21) are undergoing rigorous study for therapeutic intent, shifting the focus from MSC mediated regeneration potential toward exploiting MSC “medicinal signaling” (22). Another promising component of MSC therapy involves its natural cell-cell communication ability via exosomes, nanometer-sized lipid membrane bound vesicles that secrete a variety of cargo molecules to maintain tissue homeostasis (23). Drug encapsulation using exosomes can extend the agent's half-life, and maximize targeted and controlled delivery with minimal effects on healthy tissues (24). This can be beneficial in cases where a biologic therapeutic (e.g., cytokines, miRNA, growth factors) may either have difficulty reaching the pathologic cerebral area, or cause systemic side effects if delivered on their own (e.g., inflammatory reactions from systemic IL-2).

### Immune Cells

Immune cell delivery is the other arm of CNS cellular therapies currently being investigated, mainly in the context of treating malignancy, but more recently also being explored for neurodegenerative conditions (25). One of the primary findings of small animal studies, which established the new experimental domain of natural killer (NK) cell therapeutics, was their ability to recognize and kill human glioblastoma (GBM) cells through direct cell-mediated cytotoxicity (26). As NK cells account for only ~3% of circulating immune cells, autologous harvesting would not be able to reach therapeutic levels, and expansion *ex vivo* would be required. Many clinical trials use the immortalized NK-92 cell line, due to relative ease of expansion and implementation (compared to autologous harvesting). To circumvent the immunosuppressive environment of brain tumors, which downregulates NK activity, NK cells can be engineered *ex vivo* to overexpress activating cytokines (Il-12 or IL-15) to form “activated” NK cells, which increases tumor cell killing efficacy (27). The clinical success of chimeric antigen receptors in T-cells (CAR-T cells) for treating lymphoblastic leukemias shows promise toward implementing a similar strategy using NK cells, a lymphoid relative of T-cells, for solid brain malignancies (28). The use of CAR-T cells is an evolving form of cancer immunotherapy, in which autologously or allogeneic derived T-cells are genetically modified to target and attack specific cancer cells via chimeric antigen receptor binding (29, 30).

## Routes of Administration For Cellular Therapy

The route of administering therapeutic cells into pathologic intracerebral regions plays a critical role toward successful implantation. The BBB is a multicellular capillary network that protects the brain parenchyma from intrusion of foreign pathogens and neurotoxins, and regulates cerebral perfusion and flux of ions, hormones, and glucose to ensure normal functioning of neuronal circuits. Therapeutic cells must either circumvent the BBB or be functionalized to utilize one of many transmigratory pathways of the BBB for access to the target site (31).

Intracerebral injections offer the most direct access for cellular implantation, by bypassing the BBB. However, the stereotactic technique can carry increased risk of hemorrhage and infection (32). This method is also less appealing due to the limited effective volume of delivery, especially for larger agents (33). Intrathecal administration bypasses the BBB via injection of the therapeutic agent or cell directly into the subarachnoid space and the CSF. This approach is mainly utilized for treating leptomeningeal disease, delivering chemotherapeutics for leptomeningeal disease (34), and baclofen for analgesia (35). For parenchymal disease, intrathecal administration of cells may have limitations due to the rapid turnover of CSF (i.e., minimizing interaction time between the cell therapy and brain-CSF barrier) (31).

Vascular routes of administration include intra-arterial (IA) and intravenous (IV) administration, and are commonly employed for procedural simplicity. There was initial concern for the IV route being a potential nidus for pulmonary thromboembolism (36), but a recent systematic review of 47 randomized clinical trials utilizing intravascular administration of MSCs found no statistically significant risk of embolic complications compared to controls (37). Nevertheless, studies report injected cells accumulating within the microvasculature of the lung, i.e., a “pulmonary trapping” effect (38), leading to decreased cell engraftment at the treatment target. Intracarotid (i.e., IA) injections can bypass filtering organs and allow cells to reach the CNS to a greater degree than by IV (39).

Hyperosmolar BBB disruption involves intravascular delivery of an agent, typically mannitol, that increases oncotic pressure and drives fluid outside of microvascular epithelial cells, causing shrinkage and thus paracellular passage of therapeutics, including stem cells, into the brain (40). A drawback of this procedure is that it may lead to increases in BBB permeability in off-target brain regions. This would for example, allow greater exposure to endogenous neurotoxins (i.e., albumin), which may result in adverse effects that include vaso-vagal responses and focal seizures (31). Due to the risk of compounding vasogenic edema, using this approach to treat an entire multifocal CNS disorder that presides over separate cerebrovascular regions may not be feasible to complete in a single treatment session and may have to be divided over multiple periods (41).

Intranasal delivery is a developing administration approach that is not yet fully understood but thought to bypass the BBB by relying on migration along olfactory and trigeminal nerve tracts and into CSF flow tracts (42). Many studies have demonstrated higher CSF levels of chemotherapeutics, small molecular drugs, and nanoparticles using this innovative approach, relative to conventional intravenous routes. However, this strategy may be limited by clearance from the ciliated mucosal epithelium (43).

Implantable devices such as the Ommaya reservoir have successfully been used for delivering growth factors, analgesics and chemotherapy directly into CSF circulation. This approach has notable drawbacks that would preclude the delivery of cells due to clogging and pump failure (44). Collectively, limitations encountered by these routes may include invasiveness, low rates of engraftment, and low target region specificity. The common cell administration routes implemented in preclinical and clinical trials, each with their specific advantages and disadvantages, are summarized in Table 1.

**Table 1:** Administration routes for intracerebral cellular therapy.

**Technique**	**Advantages/Disadvantages**		**Ref**
Intraparenchymal (Stereotactic injection)	**ADV:** **DIS:**	Bypasses BBB; most direct access to CNS region of pathology, high engraftment rate Invasive (risk of hemorrhage, infection, injury to normal tissue), glial scar formation, not feasible in poor surgical candidates	(45)
Intrathecal (Injection)	**ADV:** **DIS:**	Bypasses BBB Invasive, high CSF turnover	(46)
Intrathecal Device	**ADV:** **DIS:**	Bypasses BBB Invasive, device failure (improper dosage, cell death inside reservoir, clogging)	(47)
Intranasal	**ADV:** **DIS:**	Bypasses BBB, Least invasive Poor engraftment rates, targeting, cells required to migrate long distances	(48)
Systemic (IA)	**ADV:** **DIS:**	Higher cell engraftment compared to IV Embolism risk, non-specific targeting	(39)
Systemic (IV)	**ADV:** **DIS:**	Relatively less invasive than IA Pulmonary trapping effectReticuloendothelial trapping (liver, spleen)	(49)
Hyperosmotic BBB disruption	**ADV:** **DIS:**	Higher cell engraftment compared to IV Embolism risk, non-specific targeting	(50)

## MRgFUS Technology and Clinical Applications

Choosing one of the aforementioned delivery routes is governed by specific preferences such as the required accuracy of targeting or minimizing the degree of invasiveness. The choice can also be dictated by characteristics of the CNS pathology, such as focality. A condition like PD for example has lesions typically occurring in one region compared to Multiple Sclerosis (MS) where lesions are in multiple regions. Engraftment rates of cells in some studies have not been encouraging. For example, one study investigating IV injection of MSCs into a traumatic brain injury (TBI) rat model yielded <4% of cells reaching arterial circulation (51). Therefore, a growing number of researchers have been investigating the use of focused ultrasound (FUS) under MRI guidance (MRgFUS) to pre-treat focal pathologic regions for enhancing cell delivery. MRgFUS is a non-invasive modality that is advantageous for its spatial specificity and minimal off-target effects. MRgFUS technology is gaining substantial interest for its ability to provide controlled, non-invasive, and targeted therapeutic ultrasound energy, which can be adjusted to create a variety of beneficial biological effects for treatments in the brain (52). These specific effects include destructive thermal ablation (53, 54), radiosensitization (55), immune activation (56, 57), BBB opening for therapeutic delivery (58, 59), and stem cell homing (60, 61).

The variation in induced effects is controlled by the mode of application, which can be continuous or pulsed (i.e., non-continuous), and further modified by varying the duration and intensity of the applied ultrasound energy. Thermal tissue effects predominate with continuous exposures, and temperatures can rise to 60°C in the focal region within seconds, leading to tissue destruction by the process of coagulative necrosis. With pulsed FUS (pFUS) exposures, mechanical tissue effects predominate, and temperature elevations are minimal (within the range 4–5°C) (62–64). Globally, focused ultrasound ablative therapies have been approved and implemented for thyroid nodules, bone metastases, uterine fibroids, and tumors of the liver, pancreas, kidney, prostate, and breast. With regards to intracranial applications, the FDA approved MRgFUS ablation for essential tremor in 2016, and recently tremor-dominant PD in 2018^1^ These two milestones have provided the motivation for more preclinical and clinical studies to be proposed and developed. To date, up to 2000 patients in the US have undergone MRgFUS treatments for either the aforementioned FDA-approved indications or in new clinical studies for the treatment of neuropathic pain (NCT03111277), AD (NCT04118764), epilepsy (NCT02804230), obsessive compulsive disorder (NCT03156335), ALS (NCT03321487), and brain malignancies (NCT00147056).

### FUS Mediated BBB Opening

Reliable BBB opening (BBBO) is achieved with the combination of MRgFUS and IV injection of ultrasound contrast agents (i.e., microbubbles, MBs) which are typically 1–10 micron lipid or albumin based spheres containing a bio-inert gas. Early pFUS studies without using MBs demonstrated that meaningful mechanical effects, such as those required for permeabilizing the BBB, could not be generated without the presence of tissue damage (65). A landmark study by Hynynen et al. found that incorporating MBs significantly improved the clinical feasibility of the technique. It allowed for finer control of BBB permeability and required lower intensities, lessening the risk of skull heating and damage (66). After injection, the MBs travel throughout the circulatory system and eventually reach the capillaries within the target volume of the FUS transducer.

Acoustic cavitation is one of the non-thermal pFUS based mechanisms for generating bioeffects. This occurs in the form of expansion of the MBs during the negative pressure part of the ultrasound cycle and contraction during the positive pressure part. Upon pFUS exposure, the MBs transmit these mechanical oscillations onto the endothelial cells, which can alter BBB permeability. Low pressure amplitudes, in which MB oscillations remain stable (i.e., non-inertial cavitation), are employed to induce transient BBB opening through a number of proposed paracellular and transcellular mechanisms. If the pressure amplitude becomes too high, the MBs undergo unstable oscillations (i.e., inertial cavitation) where they expand and eventually collapse. This is undesirable in BBBO where shock waves generated can damage cells of the microvasculature. Hence, monitoring for cavitation is crucial for this application of MRgFUS (67).

**Figure 1:**
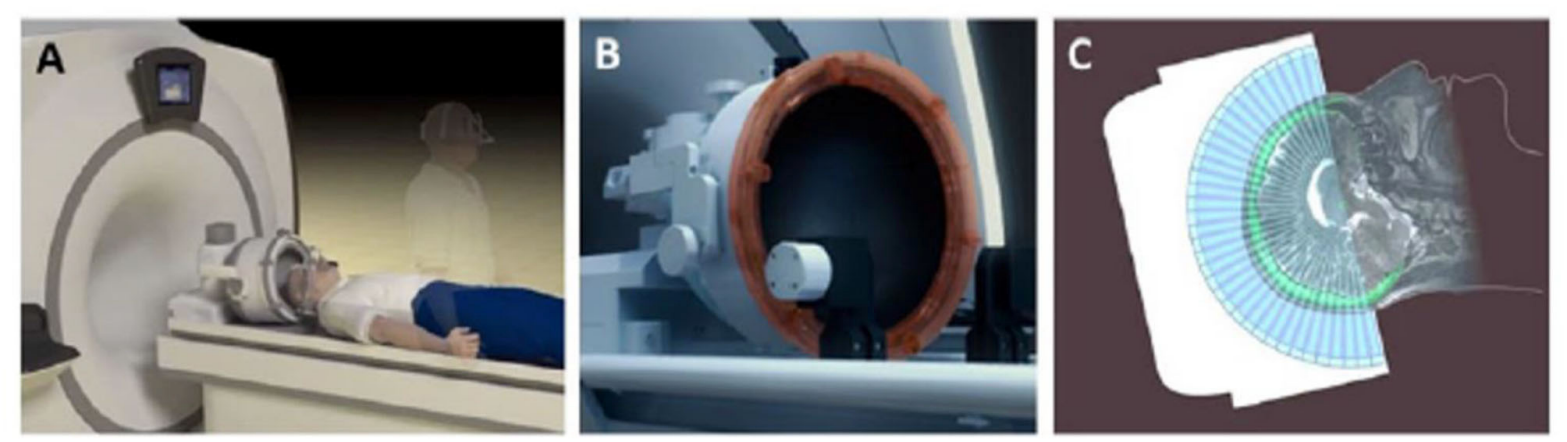
Set-up of MRgFUS patient treatment. **(A)** A schematic representation of patient lying supine on MR table being fitted with FUS phased-array transducer array; **(B)**, Close up of the 1,024 ultrasound element array for electronic steering of the ultrasound beam. **(C)** A schematic 2-dimensional representation of the multiple ultrasound beams focused non-invasively through the skull (bright green) to a single target. The image of the skull is obtained from a prior computed tomographic scan that is mechanically registered to the MR image. Information from the skull is used by the planning software to correct for aberrations to the beam paths and accurately position the focus at the desired target. Adapted from *Fishman and Frenkel, Journal of Central Nervous System Disease 2017* (68). Reprinted with permission from SAGE Publishing.

Most human MRgFUS treatments in the brain are performed using a hemispherical 1,024-element ultrasound transducer array that communicates with the MRI system (Figure 1). Each of the contiguous transducer elements is driven by an individual power source. Depending on the treatment target, specific individual elements will be activated, where the beams converge (i.e., at the “focus”), which is electronically steered in 3-dimensional space within the brain. Important to note is that the acoustic power applied to each element is typically incapable of inducing a deleterious biological effect. However, the additive power at the focus is sufficient to thermally ablate tissue, or conversely, generate the mechanical effects designed for opening the BBB. Real-time acoustic monitoring of cavitation determines optimal sonication parameters during the procedure. The transducer is fitted to the patient's head via a stereotactic frame affixed to the scalp under local anesthetic. The patient's head is coupled to the transducer via a flexible silicone membrane. The closed membrane contains degassed water for effective transmission of the ultrasound energy. The patient lies awake on the MRI table throughout the procedure and is able to respond to questions to ensure no adverse symptoms are being experienced.

Pre-procedural T1, T2 and T2^*^ images are obtained from the MRI scanner and transferred to the MRgFUS graphic user interface (GUI). The target treatment volume is identified using an overlay on the MR images. The location of the region of treatment will determine which of the 1,024 ultrasound transducer elements will be activated during treatment. The beam is then steered electronically, automatically rastering (i.e., moving from point to point) through the treatment volume with user defined spacing (69).

Immediately prior to the beginning of treatment, a suspension of MBs is then injected intravenously. Before administering full sonication treatments, a “power-ramp” test is done at each region to determine the minimal power output that opens the BBB for that specific patient. Short sonications to the region are applied incrementing in power at 5% intervals until cavitation is detected with the use of an acoustic feedback controller via hydrophone measurement. The full sonications are then delivered at 50% power of the determined cavitation threshold (70). Real time MR thermometry is employed to ensure tissue temperatures that create irreversible change in surrounding tissues are not reached during BBBO (71). After sonication treatments are complete, gadolinium enhanced T1 imaging, which has been shown to correlate to the degree of BBBO and therapeutic delivery, is obtained for verification (72). Transient BBBO with MRgFUS was successfully verified and determined to be safe in patients with Glioblastoma (NCT03551249; NCT03616860), Alzheimer's Disease (AD) (NCT04118764), and more recently Amyotrophic Lateral Sclerosis (ALS) (NCT03321487). Trials for PD (NCT04370665) and HER2 amplified brain metastases (NCT03714243) are currently underway (73–75). Additional clinical trials exploring other direct effects of BBB opening (e.g., glial cell activation, amyloid beta plaque clearance, neurogenesis) for neurodegenerative diseases (NCT03739905) are planned (76). As effects of BBB-opening have been shown to last from 4–8 h, (67) therapeutic cells could then be administered intravenously or intra-arterially within this window.

## MRgFUS-Mediated Cell Delivery

### Preclinical Studies of MRgFUS-Mediated Cell Delivery

Successful MRgFUS assisted cell delivery was first demonstrated using a combined approach of intracarotid injection of dual GFP/Iron Oxide-labeled NSCs and MRgFUS targeted to the striatum and hippocampus. The goal of this study was to demonstrate the feasibility, reversibility, and safety of this approach over conventional methods (i.e., injection and hyperosmolar BBBO). Histological analysis showed limited damage and red blood cell extravasation in non-target areas, and 32 viable NSCs per square millimeter of sonicated brain tissue, with neuronal specific biomarkers present 4–24 h after treating (77). Another study using bone marrow MSCs administered IV with FUS treatments to the lateral hippocampal area, demonstrated a 2-fold increase in engraftment rate compared to IV injection alone. FUS treatments also show increased expression of the cell adhesion molecules (CAM), including ICAM and VCAM, which is thought to improve targeting of the cells (78).

In addition to stem cells, immune cells have also been investigated using MRgFUS BBBO. CAR NK-92 cells were administered IV in a murine model of HER2-amplifed brain metastasis. Interestingly, IV injection of the NK cells immediately before BBBO resulted in a 5-fold increase in the number of cells observed to be delivered compared to injecting cells after BBBO (79). A follow-up study investigated survival in this model using temporally different pFUS treatment protocols. A “front-loaded” group, which concentrated pFUS treatments in the 1st week of treatment, was found to have greater survival relative to controls, whereas the group that had more equally distributed treatments did not show improvement (80). This finding introduced more questions to be investigated, such as how treatment frequency affects therapeutic cell delivery, or if these treatments become inefficacious as intracranial tumor burden passes a certain threshold.

**Figure 2:**
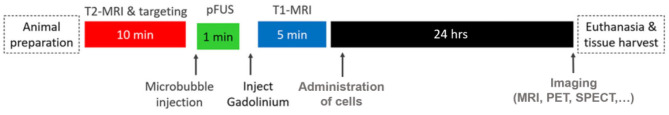
Typical timeline for an MRgFUS preclinical study investigating cellular delivery. Adapted from *Shen et al. Cell Transplantation 2017* (81). Reprinted with permission from SAGE Publishing.

**Figure 3:**
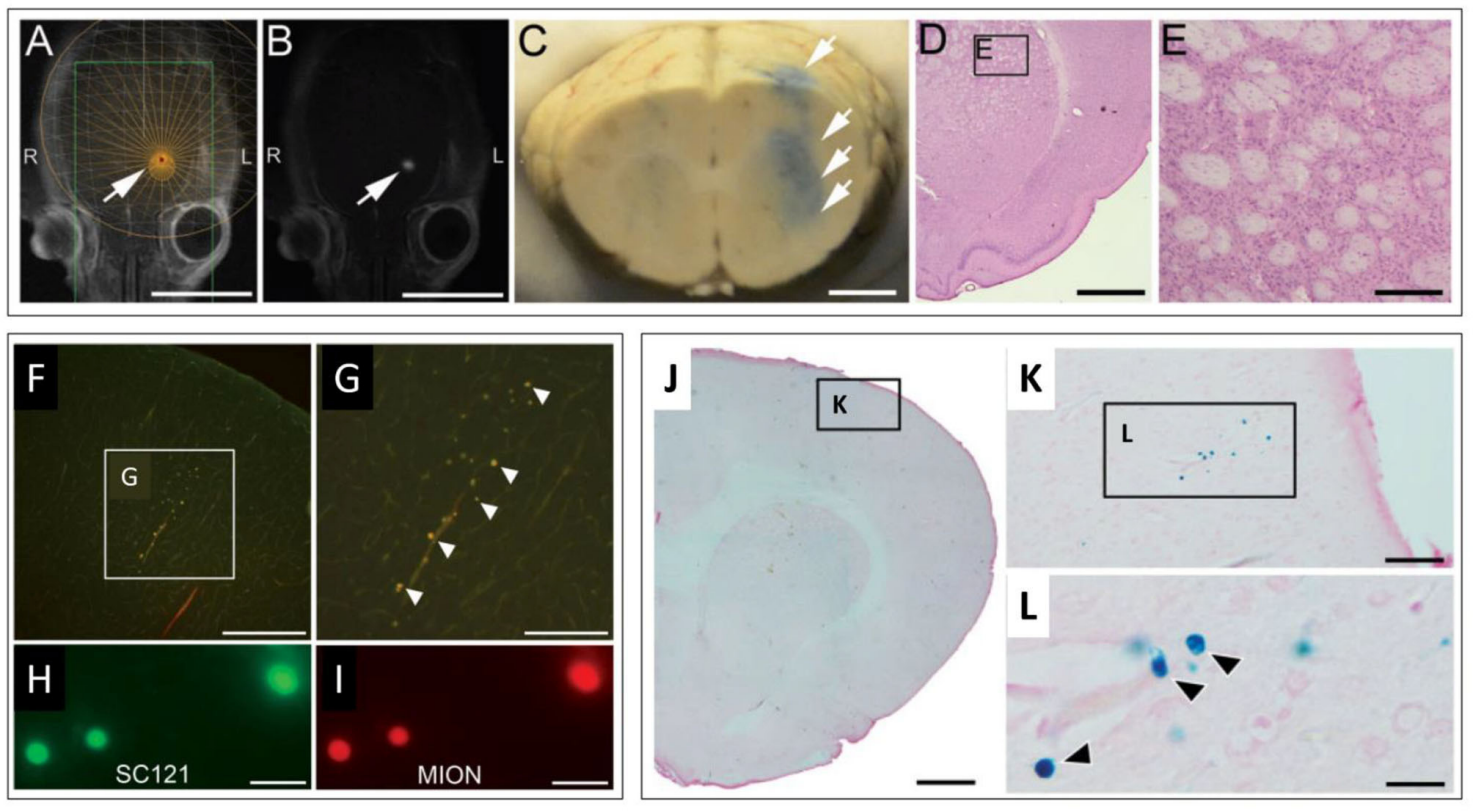
MRgFUS mediated delivery of dual labeled (fluorescence & SPION) NPCs in the rodent brain. **(A,B)** Representative screen captures from an MRgFUS system graphic user interface. **(A)** T2 weighted axial MRI image of a rat brain showing treatment target (arrow) overlay. **(B)** T1 contrast MRI image showing hyperintense signal from gadolinium extravasation at location of treatment (arrow), indicating successful BBBO. Signal coincides with treatment target in “A”. **(C)** Whole brain coronal section indicating successful BBBO, evidenced by Evans blue dye (arrows) that extravasated in the region of the focal zone. **(D)** H&E stained brightfield histological section demonstrating unaffected tissue in the region of MRgFUS treatment. **(E)** higher magnification region from “D” (inset). **(F–I)** Fluorescence microscopy images of fluorescently labeled NPCs in the brain. **(F)** Fluorescently labeled NPCs in the dorsal cortex. **(G)** higher magnification of inset in “F”. **(H)** Fluorescent signals detected from labeled human cytoplasmic antigen (SC121). **(I)** Higher magnification of fluorescently labeled NPCs. Co-localization of fluorescent signals in “H” and “I” provide evidence that labeled cells are NPCs (human). **(J–L)** Brightfield microscopy images of Prussian blue stained histological sections (for SPION) indicating the presence of NPCs. **(J)** Low magnification image. **(K)** Inset in “J.” **(L)** Inset in “K.” Individual cells (blue) are seen (arrows). Scale bars: A, B = 10 mm; C = 2 mm; D = 2 mm; E = 200 μm; F = 400 μm; G = 200 μm; H, I = 20 μm; J = 1 mm; K = 50 μm; L = 5 μm. Adapted from *Shen et al. Cell Transplantation 2017* (81). Reprinted with permission from SAGE Publishing.

Investigations evaluating the potential of magnetic enhancement of MRgFUS for cellular delivery were also conducted. Dual labeled, fluorescent/super paramagnetic iron-oxide nanoparticle (SPION), human NPCs (hNPCs) were administered following MRgFUS BBBO. The procedural timeline for this study is shown in Figure 2. Three different magnets were then evaluated, positioned in the head region of the treated rats following the injections. Increasing magnet strength was found to be correlated with higher ratios of SPION labeled hNPCs to non-SPION labeled hNPCs observed in the treated brains (Figure 3) (81). This procedure, using MRgFUS and an external magnet, was previously demonstrated for brain delivery of magnetic nanoparticles on their own (82). Whether the addition of magnetic cell labeling and targeting to a pFUS approach provides a clinically significant improvement will have to be determined.

More recently, BBBO via low intensity ultrasound was evaluated in a brain-ischemia rat model, induced by middle-cerebral artery occlusion. Although this study was limited by using unfocused low intensity ultrasound and not pFUS, the results are noteworthy in that they demonstrated significant increases in IV-administered MSC engraftment and slight improvement in neurological outcomes, compared to IV injection of the MSCs alone. The authors acknowledged that using a focused beam would allow better spatial control of delivery and treatment (83).

The results described above provide important proof-of-concept validation using MRgFUS for enhancing cellular delivery, as well as first insights into the mechanisms involved in in this process. The general consensus is that physical/structural alterations generated (i.e., gaps), such as those facilitating smaller agent delivery, are less likely to be involved due to the relatively larger size of cells, which can be orders of magnitude greater. Other potential mechanisms involved are presented in the following section (“FUS effects on cell homing”). Preclinical studies of MRgFUS assisted cell delivery for other CNS pathologies are underway. Established animal models of MS (84) or AD (76) are considered to be good candidates for future investigations. The preclinical studies employing ultrasound for enhancing cellular delivery to the brain are summarized in Table 2.

**Table 2:** Preclinical studies investigating ultrasound for cellular delivery to the brain.

**Study design and highlights**	**Year**	**Ref**
IA administration and MRgFUS increased NSC engraftment into rat striatum and hippocampus. Safety and reversibility of the treatments were also demonstrated.	2011	(77)
IV administration and MRgFUS improved delivery of NK92 cells in rat breast cancer brain metastasis model. More cells were delivered when administered prior to MRgFUS compared to afterward.Follow-up study in this model demonstrated that enhanced cellular delivery translates to improved survival. Front loading treatments compared to even temporal distribution also improved outcomes.	2013 2016	(79) (80)
IV administration and MRgFUS improved delivery of dual labeled NPCs in rat brain. Magnetic targeting of SPION labeled cells with external magnet improved retention of cells compared to non-labeled cells.	2017	(81)
IV administration and FUS pretreatment in rat brain resulted in a 2-fold increase in MSC transplantation in the lateral hippocampus. Improved delivery was apparently associated with increased expression of CAMs.	2020	(78)
Low intensity, non-FUS improved MSC engraftment 2-fold in rat brain ischemia model. Enhanced engraftment was associated with improved neurological outcomes.	2020	(83)

### FUS Effects on Cell Homing

Vascular extravasation of stem cells to sites of injury is analogous with endogenous immune cell behavior (leukocytes, monocytes, t-cells, dendritic cells) in that stem cells also follow a sequence of chemoattraction, margination, rolling, adhesion, and diapedesis. This is due to similar expression profiles of integrins, cytokine and chemokine receptors (e.g., VCAM-1, B1 integrins) (85, 86). Many preclinical studies have demonstrated in multiple organ tissues, including the CNS, that pretreatment with pulsed focused ultrasound (pFUS) non-destructively alters the vascular endothelial microenvironment, evidenced by an upregulation of chemokines, cytokines, trophic factors (CCTFs) and cell adhesion molecules (CAM). This pattern of pFUS mediated mechanical effects and biological changes permit significant increases of stem cell/immune cell homing and transmigration compared to simple vascular injection, i.e., referred to as enhanced homing, permeability & retention (EHPR) (87). In a murine skeletal muscle model, Burks et al. showed that pFUS exposures increased infiltration and presence of dual fluorescent/super paramagnetic iron oxide nanoparticles (SPION)-labeled macrophages, MSCs and endothelial progenitor cells (EPCs) relative to untreated controls. FUS exposures were shown to result an upregulation of cytokines (notably Il-1, TNF-a, IFN-y), growth factors (VEGF, SDF-1α) and cell adhesion molecules (ICAM-1 and VCAM-1) (88, 89). Similar outcomes were noted in a murine kidney model, where an 5-fold increase in bone marrow stromal cell count was noted 3 days post-treatment compared to the contralateral untreated kidney, aided by visual confirmation via T2^*^ weighted MRI and histology (87).

**Figure 4:**
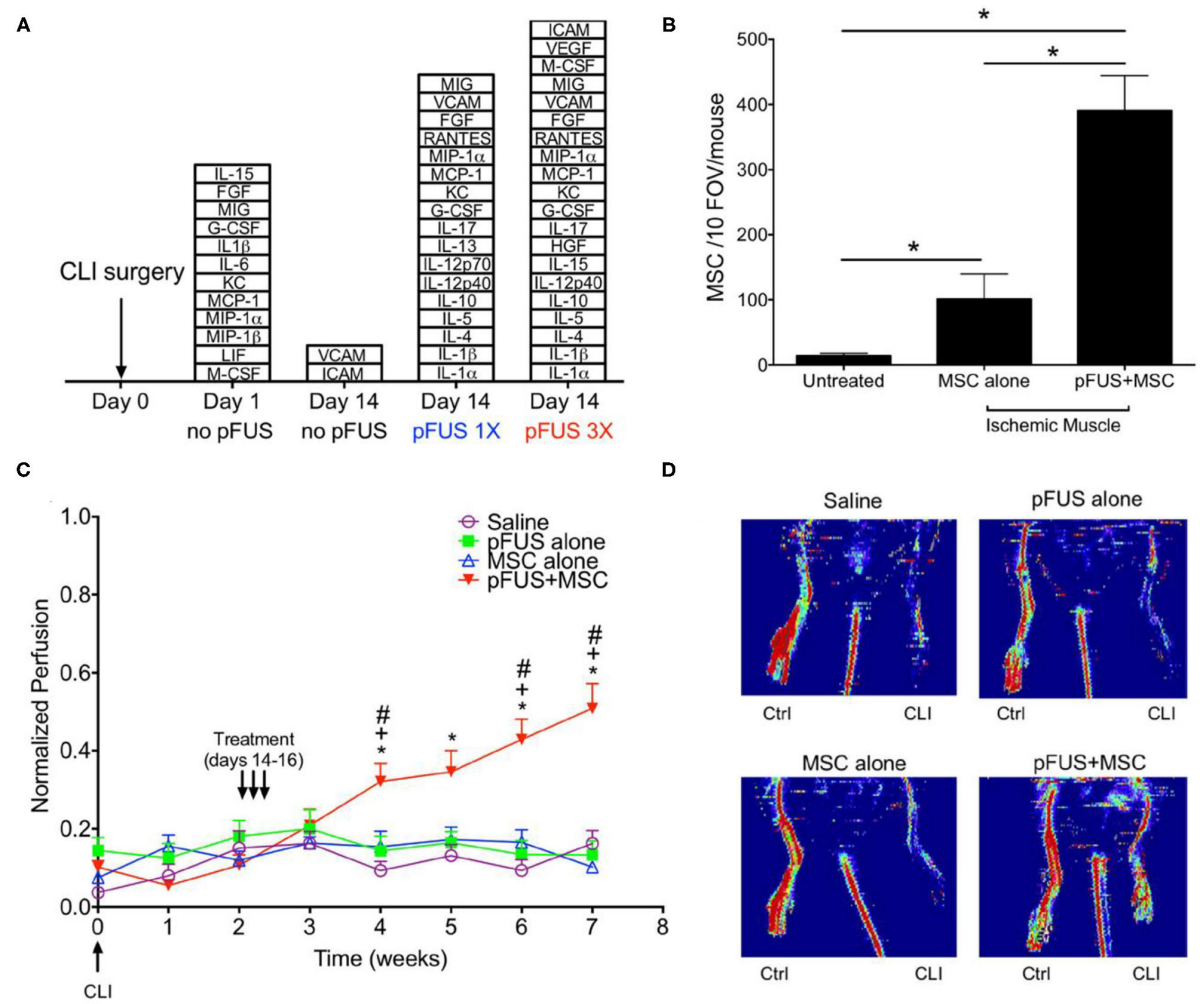
FUS mediated delivery of MSCs in murine model of CLI in skeletal muscle **(A)** Stacked box plots comparing proteomic responses of CLI muscle between FUS treatment and untreated controls. The chemokines, cytokines, trophic factors, and cell adhesion molecules listed are those have significantly higher levels than those in normal muscle after CLI alone (*n* = 6). **(B)** SPION labeled MSCs in control and FUS treated skeletal muscle in CLI mice. Significantly greater numbers of MSCs were observed in FUS treated animals, based on Prussian blue staining of cells (*n* = 5). **(C)** Temporal changes in normalized perfusion comparing control and FUS treated CLI mice. Results are based on laser Doppler perfusion imaging (LDPI) indicating reperfusion that occurred in FUS treated animals only (*n* = 7). **(D)** Representative LDPI images at week 5 in the study for each experimental group. Adapted from Tebebi et al. *Sci Rep 2017* (91). Reprinted with permission from *Nature Research*.

In subsequent studies, the same group showed how pFUS mediated delivery of MSCs improved disease outcomes. This included improved survival in a model of cisplatin induced acute kidney injury (AKI) (90), and improvement in reperfusion and a reduction in fibrosis in a model of critical limb ischemia (Figure 4) (91). Most recently, this procedure was shown to also be successful for enhancing homing to the myocardium in the left ventricle in a rat model, indicating the potential of this approach for cardiac regeneration (92). It has been proposed that FUS induces Ca influx via mechanosensitive calcium channels (TRPC1), leading to activation of the NFkβ pathway and transient expression of TNFα. The increase in TNFα then drives COX2 canonical pathways that generate cell homing signals (93, 94).

The studies described above were carried out using pFUS treatments without the use of microbubbles. Instead, higher acoustic pressures were used, being approximately 5-fold greater than those used for BBB opening. These treatments also targeted the parenchyma as opposed to the vasculature. In previous studies, the mechanical effects generated by these treatments appeared as widening of intercellular spaces and were shown to enhance interstitial transport of a range of therapeutic formulations (e.g., nanoparticles, monoclonal antibodies, plasmid DNA) in skeletal muscle (63, 95), solid tumors (96), and the brain (97). The proposed ultrasound mechanism for creating these effects is the generation of unidirectional radiation forces, which if large enough can displace tissue locally in the region of the focal zone. Through repetitive pulsing, it is thought that this movement of tissue acts on the relatively weak structural elements in the tissue, being the interfaces between individual cells (95).

MRgFUS studies have also been conducted in the brain to investigate the generation of CCTFs and CAMs for the purpose of enhancing cell homing. Kovacs et al. mapped the proteomic and transcriptomic time course of MRgFUS mediated BBBO in a murine model. Within 5 min of exposure, expression of (TNF-α, IL1α/β, IL18, IFN-y) and CAM was observed, as well as stromal derived factor (SDF1-α), a significant chemokine utilized by lymphocytes and mesenchymal stem cells, within 2 h (61). These results observed in the brain were transient and consistent with those observed in earlier studies in the kidney and skeletal muscle and underlying mechanism of NFkβ activation, indicating that the effects could potentially be beneficial for stem cell homing (87, 88, 91, 98). The molecular effects in the brain were confirmed in a recent study where similar factors over the same time course were found to be upregulated by MRgFUS (60). Overall, MRgFUS effects for BBBO have been shown to be transient and safe, without producing neuronal apoptosis or inflammation (99). To date however, these specific effects were not shown to be associated with enhanced cell homing to the brain.

## Tracking Cellular Engraftment

The ability to monitor activity of exogenous cells (migration to target region, viability, differentiation), as well as potential immunogenic or tumorigenic complications, is essential for evaluating the efficacy of CNS cellular therapies, in addition to monitoring engraftment response in the patient. Clinically relevant intracranial cell tracking modalities involve structural (MRI) and tracer-based (PET/SPECT) imaging, each of which have their own specific direct and indirect methods of cell tracking *in vivo*. Experimental studies evaluating cell labeling techniques for the purpose of tracking must verify that they do not negatively affect cell viability and/or key cellular functions (migration, division, differentiation, cytokine release). The duration and modality of monitoring will depend on a multitude of factors such as the therapeutic application (i.e., short-term immunomodulation vs. long-term cell replacement/regeneration).

### Direct and Indirect Cell Labeling Techniques

Direct cell labeling involves cells that are treated with an agent before administration, allowing them to be detectable upon reaching a threshold concentration. Direct techniques places emphasis on nanoparticle fabrication, as their large surface area and other tunable characteristics allow for greater contrast or uptake of imaging agents (100). Despite relatively simple implementation, nanoparticle cell labeling may not be able to detect viability or distinguish the labeled cells from the local cells in the milieu. Furthermore, nanoparticles can either leak out, reducing engraftment resolution, or be taken up by macrophages that ingest dead labeled cells. In a clinical context, direct techniques may be suitable for short-term tracking purposes, such as verifying engraftment post-administration (101).

Indirect cell labeling requires viral or non-viral transfection of a gene transcript into the cell that encodes a reporter protein that generates a detectable signal based off of its interactions with an administered contrast/tracer agent (e.g., molecular trapping, enzymatic cleavage, or cell surface receptor interaction) (102). Since the reporter persists as long as the engrafted cells are alive, indirect techniques can distinguish cell viability, and grafted cells can be imaged repeatedly when the need arises for follow-up imaging. Since viral transfection of reporter genes carry a mutagenic risk, other genetic engineering methods such as non-viral vectors (e.g., cationic nanoparticles), or site-specific genome editing (i.e. CRISPR-cas9 delivery) could be potential viable alternatives.

### MRI-Based Techniques

**Figure 5:**
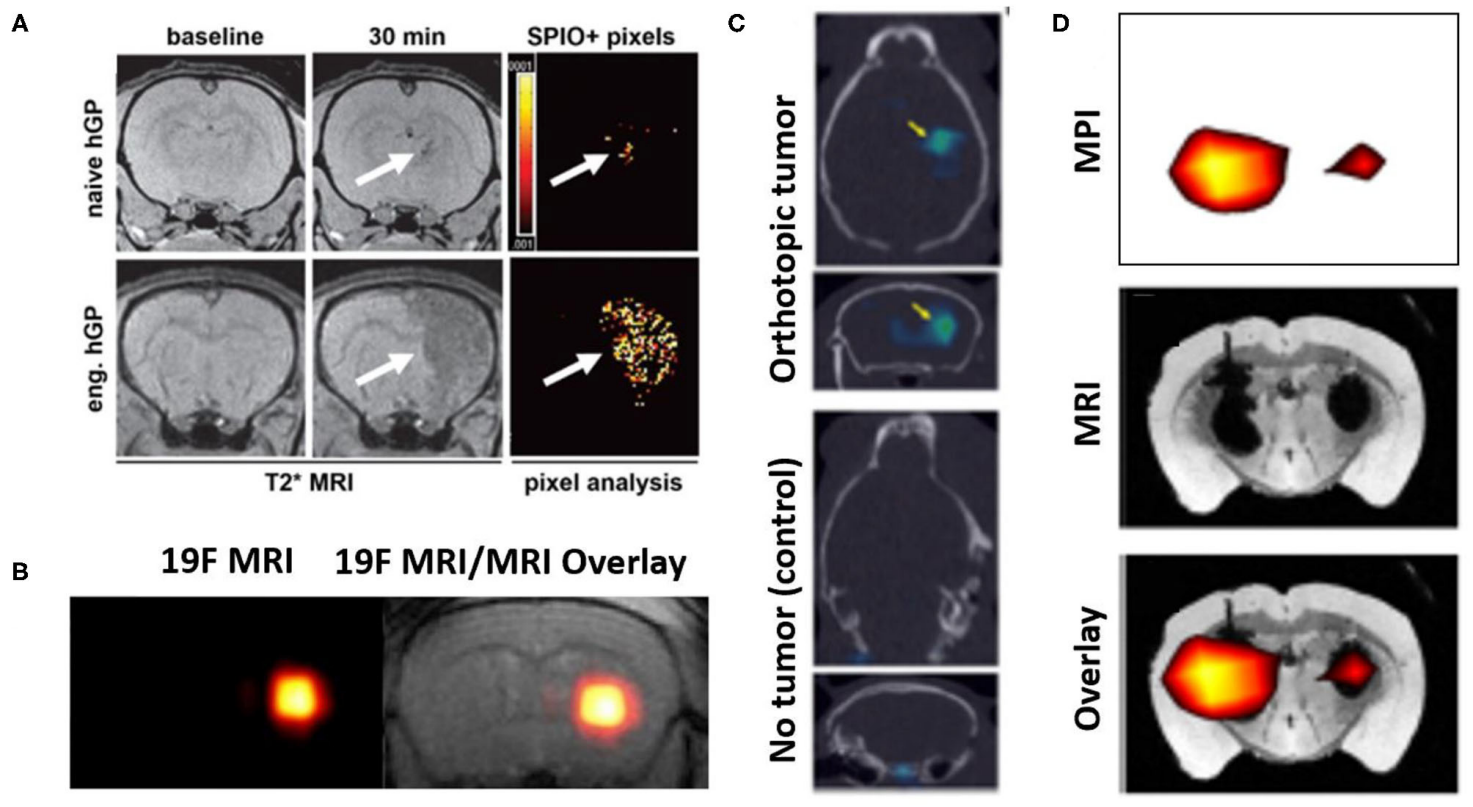
Representative examples of image-based cell tracking methods in the brain. **(A)** T2* MR images of rat brains with experimentally induced inflammation and infused with SPION-labeled human glial precursor cells (hGP). At 30 min. post-infusion, greater numbers of targeted (eng.) cells are observed in the inflamed tissue compared to naïve cells (arrows, hypointense signal). The results are supported by pixel-by-pixel analysis, comparing pre- and post-infusion MR images, which can be quantitatively compared. Modified, with permission, from “*Gorelik et al*. Use of MR cell tracking to evaluate targeting of glial precursor cells to inflammatory tissue by exploiting the very late antigen-4 docking receptor. *Radiology* 2012; 265: 175-185” (105). **(B)** (left) 19F MRI and (right) 19F MRI & T2 weighted MR images of fluorine-19 labeled glial progenitor cells injected into a mouse brain striatum. “Hot spot” in each image is clearly identified, indicating the presence of the labeled cells. Figure adapted from *Richard et al. Stem Cells Translational Medicine 2019*. Reprinted under creative commons license (106). **(C)** SPECT signals (arrows) from 111-In labeled NSCs administered into control mice (lower) and those with a glioma model (upper), to which the cells have homed. In both panels, SPECT images are overlayed on CT scans. Figure adapted from *Cheng et al. 2016* (107). This research was originally published in the Journal of Nuclear Medicine. **(D)** MPI imaging of SPION labeled MSCs in the left hemisphere (1 × 10^5^ cells) and right hemisphere (5 × 10^4^ cells) transplanted in a mouse brain. Lower panel shows MPI signals (upper panel) superimposed on T2* MR image (middle panel). Figure adapted from *Bulte et al. Tomography 2015* (108). Reprinted under creative commons license.

High soft-tissue resolution of MRI allows cellular grafts to be identified precisely within intracerebral regions. SPION-based cell tracking, which demarcates engrafted cells through changes in T2 relaxivity, is a common direct labeling method. The 1st generation of SPIONS (Feridex, Endorem), currently only available for preclinical studies in the US, was first reported in a brain trauma patient for tracking cell migration for a temporal lobe injection of autologous NSCs labeled with Feridex (103). Second generation agents (Ferumoxytol, Ferumoxtran) require cell transfection techniques (e.g., magnetoporation, magnetoelectroporation) due to less efficient uptake by cells (102). Accurate signal quantification from this iron-based labeling agent can be compromised by a number of factors, including resident macrophages engulfing SPION containing cell fragments, dilution of SPION concentration as the therapeutic cells divide, and not being able to be distinguish the cell signals from areas of hemorrhage or trauma (104). Advanced dynamic image processing techniques, such as pixel-to-pixel analysis, have demonstrated how labeling cells with SPIONs can enable monitoring cellular delivery in real-time (Figure 5A) (105).

19F MRI is another direct method which involves direct spin detection of the biologically safe isotope fluorine-19, enabling highly sensitive and quantitative “hot-spot” imaging as seen with PET/SPECT studies. One study demonstrated labeling of intracerebrally administered natural killer cells with fluorine-19, which importantly showed no cytotoxicity and change in NK cell therapeutic efficacy (109). In another study, 19F labeling of glial-progenitor cells transplanted into an ALS animal model was not found alter capacity for astrocyte differentiation (Figure 5B) (106). Indirect cell labeling for MRI has been explored preclinically, however to a lesser extent than with PET/SPECT. Overexpression of ferritin transporters can increase iron-based signaling of transplanted cells, however sensitivity in the setting of inflammation may be low (110). One group looked at transfecting a biotinylated cell surface receptor that can produce detectable MRI signal upon exposure to magnetic nanoparticle- or Gadolinium-labeled streptavidin molecules (111).

### PET/SPECT-Based Techniques

Highly sensitive “hot spot” signaling in Positron Emission Tomography (PET) and Single-Photon Emission Computed Tomography (SPECT) imaging, ensures that visualized signals are coming from the cells that were delivered. The half-lives (HL) of radionuclide agents available for direct labeling should be taken into consideration when specifying the required duration of tracking of the delivered cells. Indium-Tropolone labeling of MSCs has been shown to have no effect on proliferation or differentiation (112), but this was not the case when using Indium-Oxine (113). Technitium-99, with a HL of 6 h, was used to label neural progenitor cells that carried gene delivery products to a mouse glioma model (114). Another SPECT study tracking NSC homing to glioblastoma tumors in mice used mesoporous silica nanoparticles conjugated with Indium-111 (HL: 67 h). The use of this agent formulation lowered the required dosage of the radionuclide and thus lessened chances of cellular damage (Figure 5C) (107). In PET imaging, fluorine-18 radioisotope (HL: 110 min) was used to track MSCs and multipotent adult progenitor cells (MAPCs) and were shown not affect the main cell characteristics (115). Bioluminescent and fluorescent imaging methods have also been used in rodent studies to track NSCs to study neurodegenerative disease, where results of these agents corroborated the results of the PET/SPECT imaging (116).

### Magnetic Particle Imaging

Magnetic particle imaging (MPI) is a developing modality that involves applying external magnetic fields (i.e., MRI) to directly detect exogenously administered SPIONs (Figure 5D) (108). SPIONs in the context of MPI behave as tracer agents, producing “hot spot” signaling as seen with F-19 MRI or PET/SPECT. While still in its infancy, MPI technology potentially has additional applications in guiding hyperthermia therapy, making physiological measurements in cerebral and cardiac vasculature, and assisting with diagnosis of acute stroke (117). Intracerebrally injected MSCs labeled with 1st generation SPIO agents (Feridex) and MSCs labeled with 2nd generation agents (Ferumoxytol, Ferucarbotran) injected into mouse calvarial defects were tracked using MPI as initial proof-of concept investigations (108, 118). This technique has been shown, for example, to produce excellent correlation between the “hot spot” signal generated and the number of cells being imaged (108). Instrumentation suitable for human use is currently in development. MRgFUS cell delivery could potentially benefit from the use of a hybrid MRI/MPI scanner, where MRI would delineate areas of FUS exposure and MPI-cell tracking verify that the cells have reached their target region (119, 120).

## Potential For Clinical Translation

CNS pathologies throughout the diagnostic spectrum may vary in their progression, effects on BBB integrity, and subsequent cellular and inflammatory responses. Clinical presentations may overlap and involve mass effect symptoms (headache/nausea), excitotoxicity (seizure, akathisia), neuropathy, focal deficits in cognition, motor, sensory, gait, coordination, possible behavioral changes, and at its worst, the inability to perform life-sustaining functions. For a given CNS condition, the selection and modification of the therapeutic cell type is informed by the symptoms presented and the desired mechanism of action (i.e., cell-mediated cytotoxicity, recruiting other cells, differentiation, replacement, immunomodulation, secretion, prodrug conversion). By employing the advantages of increased target specificity and cell homing and retention using pFUS, cellular therapy applications in the brain may obtain better clinical outcomes. The following section will outline potential benefits of employing MRgFUS delivery approaches for certain CNS disorders.

### Neurodegenerative Diseases

Neurodegenerative conditions are expected to become more prevalent in the years to come as life expectancy continues to increase. PD alone is projected to affect 14 million people world-wide by 2040 (121). Pharmacological therapies may only treat or temporarily delay severe symptoms (e.g., acetylcholinesterase inhibitors for AD, riluzole for ALS). New cell therapy strategies are under consideration for treating underlying pathology, through mechanisms such as functional cell replacement, enhancing immune system functions to clear aggregated proteins, or modifying microenvironments through growth factor delivery (4–7). Strategies for PD that employ cell replacement therapies are considered promising for restoring dopaminergic neurotransmission in order to functionally rescue the dopamine-depleted striatum (122). Functional recovery has been achieved in murine models of Huntington's Disease via MSCs overexpressing brain-derived neurotrophic growth factor (BDNF) injection (4). as well as in ALS models using glial cell derived neurotrophic factor (GDNF)-secreting NPCs via intracranial injection (123). Clinical investigations involving intra-putaminal injections of ESC-derived dopaminergic neurons are currently ongoing (124). MRgFUS can be used to accurately target pathological regions associated with this class of diseases (i.e., the striatum in PD, caudate nucleus in HD, motor cortex in ALS) in lieu of stereotactic injection and increase therapeutic cell homing. MRgFUS has been shown to be safe for repeated treatment; an obvious advantage over direct stereotactic injections (125).

### Malignancies

MRgFUS cell therapies can potentially play a role in post-glioma resection consolidation therapy and treatment of brain metastases. Recurrence remains a significant issue in the management of glioblastoma (GBM), a devastating condition with a 12–18-month median survival (126). This may be due to residual tumor cells developing resistance to chemotherapeutics, one mechanism which involves efflux transporter upregulation of the BBB surrounding the glial tumor (127). iNSCs expressing tumoricidal molecules (128), and MSCs that release exosomes containing anti-cancer miRNA (129), both have been shown to reduce glioma growth rates and prolong median survival in mouse models. NSCs overexpressing cytosine deaminase, the enzyme converter of prodrug 5-FC to 5-FU, was demonstrated to be safe in a study of 15 patients with high-grade gliomas post-resection (130). FUS-mediated delivery of NK-92 cells for a HER2-amplified brain metastases rat model showed increased survival (80). Repeated MRgFUS BBBO treatments in GBM patients was deemed safe in a recent 2020 study (131). This highlights the potential that anti-cancer cell therapies delivered this way can be done using multiple treatments.

### Autoimmune Diseases

Multiple Sclerosis involves autoimmune destruction of the white matter tracts throughout the CNS which follows an unpredictable spatiotemporal progression. Because of its multifocal nature, the therapeutic approach of a MRgFUS stem-cell delivery regiment may be beneficial for disease modifying therapies of MS or for treating acute flares, while other routes such as direct injection may be less optimal (132). Since the clinical management of an MS flare already involves MRI scanning to detect new lesions of immune hyperactivity, an MRgFUS procedure to deliver immunomodulatory MSCs or NSCs to these regions could be seamlessly integrated (133). By combining cell therapies with standard of care, which involve systemic high potency corticosteroids and a tapered course of oral corticosteroids, the high burden of these medications that confer adverse effects (i.e., Cushing's syndrome symptoms of weight gain, impaired wound healing, muscle breakdown) may be reduced (134). Multiple clinical trials examining MSC administration via intrathecal (46) or intravenous routes (135, 136) have demonstrated functional improvement. Intrathecal MSC administration was tested in 10 patients with medication refractory MS showed increased proportion of regulatory T-cells within 24 h and mean Expanded Disability Status Scale (EDSS) scores dropping from 6.7 to 5.9 at 3 months (137).

### Acute CNS Pathologies

Overlapping cell signaling pathways and mechanisms that activate cell death and neuroprotective mechanisms exist between ischemic stroke and TBI (138). In ischemic stroke, prolonged hypoxia of a CNS region secondary to either thrombotic or embolic occlusion, or global hypoperfusion, leads to necrotic and apoptotic cell death and edema following protease-mediated breakdown of the BBB (127). TBI, while acute in onset, imparts a poor prognosis from its chronic sequelae, characterized by mitochondrial dysfunction, metabolic disturbances, glial cell over-activation, excitotoxicity, and vasospasm. Cell therapies for these conditions are aimed at preventing further neuronal death through promoting angiogenesis, downregulating inflammation, and increasing synaptic plasticity. Recent results from an open-label phase IIa trial of stereotactic injection of the SB623 line (genetically modified allogeneic MSCs expressing an intracellular domain of the notch signaling pathway, involved in neuronal differentiation), showed that 16 of 18 patients with a 6–60 month stroke history exhibited significant improvements in European Stroke Scale and NIH Stroke Scale scoring (139). Using an MRgFUS BBBO approach to pretreat the ischemic penumbral region or the mechanically impacted parenchyma before intravascular delivery of cells could potentially be beneficial. Currently, only one preclinical study examining this approach for either stroke or TBI has been conducted (83).

## Conclusions

With significant improvements in FDA-approved cell manufacturing practices, rapidly accumulating clinical trial results supporting the safety of MRgFUS-mediated BBBO, and the report of studies demonstrating the potential of delivering chemotherapeutics, antibodies, drug-loaded-NPs across the BBB with this modality, it is foreseeable that neuro-interventionalists will be interested in the delivery of therapeutic cells using this method as the best next step (140). Before adapting this cell delivery approach for clinical trials, ongoing animal studies will need to continue to explore both practical and mechanistic questions that probe the process of MRgFUS assisted cell delivery. Although it has been shown that IA routes can be more effective than IV routes (141), experimental groups in future studies should include both methods of cell administration with MRgFUS to provide greater translational insight. The distribution and iteration of pFUS exposures, as well as many other sonication parameters, should be further explored and tailored to each CNS disease for safety and efficacy purposes. For instance, Alkins et al. showed that front-loading pFUS-mediated NK-92 cell treatments to the HER2 amplified brain tumor mouse, rather than distributing them over time, improved survival outcomes (80).

**Figure 6:**
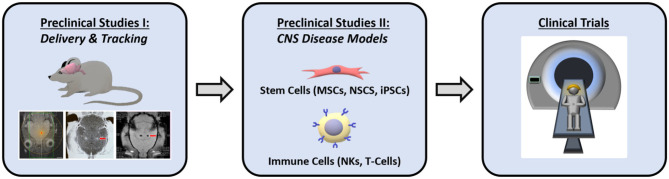
Schematic representation of the anticipated preclinical and clinical stages involved for clinical translation in using MRgFUS for enhancing cellular therapy.

In conclusion, the question of whether an MRgFUS assisted cell delivery approach can significantly reduce neurological disease morbidity/mortality, and hospital costs (e.g., surgically re-intervening on patients with recurrent GBM), is being discussed as FUS technology undergoes widespread adoption. The continued investigation of MRgFUS technology for this specific therapeutic purpose will be aided by many more preclinical studies combining different cell types with tracking methods. Overall, the results from these studies are encouraging and provide motivation to further pursue this application and evaluate its feasibility for clinical translation (Figure 6).

More recently, it was announced that MRgFUS ablation for tremor-dominant Parkinson's will receive Medicare coverage. Intracranial MRgFUS treatments can be carried out with any MRI with a modified table to house the hemispherical transducer array and currently, there are 800 systems in the US in operation (See footnote 1), and many more worldwide. As clinical trials exploring one-time stereotactic injections of stem cell therapies are ongoing and show promise, the aspects of repeatability and non-invasiveness of MRgFUS delivery will enable greater inclusion of patients who may be poor surgical candidates. The combination of cellular therapeutics and MRgFUS mediated delivery shows great potential for helping to usher in the next generation of treatment paradigms for CNS disorders.

## Author Contributions

NA, DG, EM, and VF contributed to the conception and design of the manuscript. NA wrote the first completed draft. DG, EM, and VF provided critical reviews of the first, second, and third versions of the manuscript. All authors read and approved the final submitted version of the manuscript.

## Conflict of Interest

The authors declare that the research was conducted in the absence of any commercial or financial relationships that could be construed as a potential conflict of interest.

## Footnotes

^1^https://www.fusfoundation.org/.

## References

[1] Piccini P Brooks DJ Bjorklund A Gunn RN Grasby PM Rimoldi O. Dopamine release from nigral transplants visualized *in vivo* in a Parkinson's patient. Nat Neurosci. (1999) 2:1137–40. 10.1038/16060 10570493

[2] Bachoud-Lévi A-C Rémy P Nǵuyen J-P Brugières P Bourdet C Baudic S., Motor and cognitive improvements in patients with Huntington's disease after neural transplantation. Lancet. (2000) 356:1975–9. 10.1016/S0140-6736(00)03310-9 11130527

[3] Keene CD Chang RC Leverenz JB Kopyov O Perlman S Hevner RF. A patient with Huntington's disease and long-surviving fetal neural transplants that developed mass lesions. Acta Neuropathol. (2009) 117:329–38. 10.1007/s00401-008-0465-0 19057918 PMC2676786

[4] Pollock K Dahlenburg H Nelson H Fink KD Cary W Hendrix K. MSCs genetically engineered to overexpress BDNF improve outcomes in Huntington's disease mouse models. Mol Ther. (2016) 24:965–77. 10.1038/mt.2016.12 26765769 PMC4881765

[5] Aboody KS Najbauer J Metz MZ D'Apuzzo M Gutova M Annala AJ. Neural stem cell-mediated enzyme/prodrug therapy for glioma: preclinical studies. Sci Transl Med. (2013) 5:184ra159. 10.1126/scitranslmed.3005365 23658244 PMC3864887

[6] Burger MC Zhang C Harter PN Romanski A Strassheimer F Senft C. CAR-engineered NK cells for the treatment of glioblastoma: turning innate effectors into precision tools for cancer immunotherapy. Front Immunol. (2019) 10:2683. 10.3389/fimmu.2019.02683 31798595 PMC6868035

[7] Greco R Oliveira G Stanghellini MT Vago L Bondanza A Peccatori J. Improving the safety of cell therapy with the TK-suicide gene. Front Pharmacol. (2015) 6:95. 10.3389/fphar.2015.00095 25999859 PMC4419602

[8] Takahashi K Tanabe K Ohnuki M Narita M Ichisaka T Tomoda K. Induction of pluripotent stem cells from adult human fibroblasts by defined factors. Cell. (2007) 131:861–72. 10.1016/j.cell.2007.11.019 18035408

[9] Kim TW Koo SY Studer L. Pluripotent stem cell therapies for Parkinson disease: present challenges and future opportunities. Front Cell Dev Biol. (2020) 8:729. 10.3389/fcell.2020.00729 32903681 PMC7438741

[10] Staerk J Dawlaty MM Gao Q Maetzel D Hanna J Sommer CA. Reprogramming of human peripheral blood cells to induced pluripotent stem cells. Cell Stem Cell. (2010) 7:20–4. 10.1016/j.stem.2010.06.002 20621045 PMC2917234

[11] Chang EA Jin SW Nam MH Kim SD. Human induced pluripotent stem cells: clinical significance and applications in neurologic diseases. J Korean Neurosurg Soc. (2019) 62:493–501. 10.3340/jkns.2018.0222 31392877 PMC6732359

[12] Mitsiadis TA Barrandon O Rochat A Barrandon Y De Bari. C. Stem cell niches in mammals. Exp Cell Res. (2007) 313:3377–85. 10.1016/j.yexcr.2007.07.027 17764674

[13] Takagi Y. History of neural stem cell research and its clinical application. Neurol Med Chir (Tokyo). (2016) 56:110–124. 10.2176/nmc.ra.2015-0340 26888043 PMC4791305

[14] Christian KM Song H Ming GL. Functions and dysfunctions of adult hippocampal neurogenesis. Annu Rev Neurosci. (2014) 37:243–62. 10.1146/annurev-neuro-071013-014134 24905596 PMC5531058

[15] Vieira MS Santos AK Vasconcellos R Goulart VAM Parreira RC Kihara AH. NSC differentiation into mature neurons: Mechanisms of regulation and biotechnological applications. Biotechnol Adv. (2018) 36:1946–70. 10.1016/j.biotechadv.2018.08.002 30077716

[16] Mooney R Hammad M Batalla-Covello J Abdul Majid A Aboody KS. Concise review: NSC-mediated targeted cancer therapies. Stem Cells Transl Med. (2018) 7:740–7. 10.1002/sctm.18-0003 30133188 PMC6186269

[17] Darvishi M Tiraihi T Mesbah-Namin SA Delshad A Taheri T. Motor neuron transdifferentiation of neural stem cell from adipose-derived stem cell characterized by differential gene expression. Cell Mol Neurobiol. (2017) 37:275–89. 10.1007/s10571-016-0368-x 27107758 PMC11482063

[18] Rosati J Ferrari D Altieri F Tardivo S Ricciolini C Fusilli C. Establishment of stable iPS-derived human neural stem cell lines suitable for cell therapies. Cell Death Dis. (2018) 9:937. 10.1038/s41419-018-0990-2 30224709 PMC6141489

[19] Matsushita T Kibayashi T Katayama T Yamashita Y Suzuki S Kawamata J. Mesenchymal stem cells transmigrate across brain microvascular endothelial cell monolayers through transiently formed inter-endothelial gaps. Neurosci Lett. (2011) 502:41–5. 10.1016/j.neulet.2011.07.021 21798315

[20] Reagan MR Kaplan DL. Concise review: mesenchymal stem cell tumor-homing: detection methods in disease model systems. Stem Cells. (2011) 29:920–7. 10.1002/stem.645 21557390 PMC4581846

[21] Paul G Anisimov SV. The secretome of mesenchymal stem cells: potential implications for neuroregeneration. Biochimie. (2013) 95:2246–56. 10.1016/j.biochi.2013.07.013 23871834

[22] Caplan AI. Mesenchymal stem cells: time to change the name! *Stem Cells Transl Med*. (2017) 6:1445–51. 10.1002/sctm.17-0051 PMC5689741 28452204

[23] Lai RC Yeo RW Lim SK. Mesenchymal stem cell exosomes. Semin Cell Dev Biol. (2015) 40:82–8. 10.1016/j.semcdb.2015.03.001 25765629

[24] Aleynik A Gernavage KM Mourad YSH Sherman LS Liu K Gubenko YA. Stem cell delivery of therapies for brain disorders. Clin Transl Med. (2014) 3:24. 10.1186/2001-1326-3-24 25097727 PMC4106911

[25] Earls RH Menees KB Chung J Gutekunst CA Lee HJ Hazim MG. NK cells clear alpha-synuclein and the depletion of NK cells exacerbates synuclein pathology in a mouse model of alpha-synucleinopathy. Proc Natl Acad Sci U S A. (2020) 117:1762–71. 10.1073/pnas.1909110117 31900358 PMC6983411

[26] Castriconi R Daga A Dondero A Zona G Poliani PL Melotti A. NK cells recognize and kill human glioblastoma cells with stem cell-like properties. J Immunol. (2009) 182:3530–9. 10.4049/jimmunol.0802845 19265131

[27] Fares J Fares MY Fares Y. NK cells in the brain tumor microenvironment: defining a new era in neuro-oncology. Surg Neurol Int. (2019) 10:43. 10.25259/SNI-97-2019 31528381 PMC6743677

[28] Mehta RS Rezvani K. Chimeric antigen receptor expressing natural killer cells for the immunotherapy of cancer. Front Immunol. (2018) 9:283. 10.3389/fimmu.2018.00283 29497427 PMC5818392

[29] Maude SL Laetsch TW Buechner J Rives S Boyer M Bittencourt H. Tisagenlecleucel in children and young adults with B-cell lymphoblastic leukemia. N Engl J Med. (2018) 378:439–48. 10.1056/NEJMoa1709866 29385370 PMC5996391

[30] Schuster SJ Bishop MR Tam CS Waller EK Borchmann P McGuirk JP. Tisagenlecleucel in adult relapsed or refractory diffuse large B-cell lymphoma. N Engl J Med. (2019) 380:45–56. 10.1056/NEJMoa1804980 30501490

[31] Hersh DS Wadajkar AS Roberts N Perez JG Connolly NP Frenkel V. Evolving drug delivery strategies to overcome the blood brain barrier. Curr Pharm Des. (2016) 22:1177–93. 10.2174/1381612822666151221150733 26685681 PMC4900538

[32] Chen CC Hsu PW Erich Wu TW Lee ST Chang CN Wei KC. Stereotactic brain biopsy: single center retrospective analysis of complications. Clin Neurol Neurosurg. (2009) 111:835–9. 10.1016/j.clineuro.2009.08.013 19765887

[33] Wolak DJ Thorne RG. Diffusion of macromolecules in the brain: implications for drug delivery. Mol Pharm. (2013) 10:1492–504. 10.1021/mp300495e 23298378 PMC3646902

[34] Kapke JT Schneidewend RJ Jawa ZA Huang CC Connelly JM Chitambar CR. High-dose intravenous methotrexate in the management of breast cancer with leptomeningeal disease: Case series and review of the literature. Hematol Oncol Stem Cell Ther. (2019) 12:189–93. 10.1016/j.hemonc.2019.08.008 31629723

[35] Penn RD Savoy SM Corcos D Latash M Gottlieb G Parke B. Intrathecal baclofen for severe spinal spasticity. N Engl J Med. (1989) 320:1517–21. 10.1056/NEJM198906083202303 2657424

[36] Sugiyama Y Sato Y Kitase Y Suzuki T Kondo T Mikrogeorgiou A. Intravenous administration of bone marrow-derived mesenchymal stem cell, but not adipose tissue-derived stem cell, ameliorated the neonatal hypoxic-ischemic brain injury by changing cerebral inflammatory state in rat. Front Neurol. (2018) 9:757. 10.3389/fneur.2018.00757 30254603 PMC6141968

[37] Thompson M Mei SHJ Wolfe D Champagne J Fergusson D Stewart DJ. Cell therapy with intravascular administration of mesenchymal stromal cells continues to appear safe: an updated systematic review and meta-analysis. EClinicalMedicine. (2020) 19:100249. 10.1016/j.eclinm.2019.100249 31989101 PMC6970160

[38] Fischer UM Harting MT Jimenez F Monzon-Posadas WO Xue H Savitz SI. Pulmonary passage is a major obstacle for intravenous stem cell delivery: the pulmonary first-pass effect. Stem Cells Dev. (2008) 18:683–92. 10.1089/scd.2008.0253 19099374 PMC3190292

[39] Lundberg J Sodersten E Sundstrom E Le Blanc K Andersson T Hermanson O. Targeted intra-arterial transplantation of stem cells to the injured CNS is more effective than intravenous administration: engraftment is dependent on cell type and adhesion molecule expression. Cell Transplant. (2012) 21:333–43. 10.3727/096368911X576036 21669035

[40] Gonzales-Portillo GS Sanberg PR Franzblau M Gonzales-Portillo C Diamandis T Staples M. Mannitol-enhanced delivery of stem cells and their growth factors across the blood-brain barrier. Cell Transplant. (2014) 23:531–9. 10.3727/096368914X678337 24480552 PMC4083632

[41] Bellavance MA Blanchette M Fortin D. Recent advances in blood-brain barrier disruption as a CNS delivery strategy. AAPS J. (2008) 10:166–77. 10.1208/s12248-008-9018-7 18446517 PMC2751463

[42] Li YH Feng L Zhang GX Ma CG. Intranasal delivery of stem cells as therapy for central nervous system disease. Exp Mol Pathol. (2015) 98:145–51. 10.1016/j.yexmp.2015.01.016 25645932

[43] Erdo F Bors LA Farkas D Bajza A Gizurarson S. Evaluation of intranasal delivery route of drug administration for brain targeting. Brain Res Bull. (2018) 143:155–70. 10.1016/j.brainresbull.2018.10.009 30449731

[44] Shoichet MS Winn SR. Cell delivery to the central nervous system. Adv Drug Delivery Rev. (2000) 42:81–102. 10.1016/S0169-409X(00)00055-7 10942816

[45] Zhang G Li Y Reuss JL Liu N Wu C Li J. Stable intracerebral transplantation of neural stem cells for the treatment of paralysis due to ischemic stroke. Stem Cells Transl Med. (2019) 8:999–1007. 10.1002/sctm.18-0220 31241246 PMC6766600

[46] Harris VK Stark J Vyshkina T Blackshear L Joo G Stefanova V. Phase I trial of intrathecal mesenchymal stem cell-derived neural progenitors in progressive multiple sclerosis. EBioMedicine. (2018) 29:23–30. 10.1016/j.ebiom.2018.02.002 29449193 PMC5925446

[47] Fauzi AA Suroto NS Bajamal AH Machfoed MH. Intraventricular transplantation of autologous bone marrow mesenchymal stem cells via ommaya reservoir in persistent vegetative state patients after haemorrhagic stroke: report of two cases and review of the literature. J Stem Cells Regen Med. (2016) 12:100–4. 10.46582/jsrm.1202014 28096634 PMC5227101

[48] Reitz M Demestre M Sedlacik J Meissner H Fiehler J Kim SU. Intranasal delivery of neural stem/progenitor cells: a noninvasive passage to target intracerebral glioma. Stem Cells Transl Med. (2012) 1:866–73. 10.5966/sctm.2012-0045 23283548 PMC3659670

[49] Janowski M Walczak P Date I. Intravenous route of cell delivery for treatment of neurological disorders: a meta-analysis of preclinical results. Stem Cells Dev. (2010) 19:5–16. 10.1089/scd.2009.0271 19951197

[50] Okuma Y Wang F Toyoshima A Kameda M Hishikawa T Tokunaga K. Mannitol enhances therapeutic effects of intra-arterial transplantation of mesenchymal stem cells into the brain after traumatic brain injury. Neurosci Lett. (2013) 554:156–61. 10.1016/j.neulet.2013.08.058 24016413

[51] Harting MT Jimenez F Xue H Fischer UM Baumgartner J Dash PK. Intravenous mesenchymal stem cell therapy for traumatic brain injury. J Neurosurg. (2009) 110:1189–97. 10.3171/2008.9.JNS08158 19301973 PMC2889620

[52] Miller DB O'Callaghan JP. New horizons for focused ultrasound (FUS) - therapeutic applications in neurodegenerative diseases. Metabolism. (2017) 69S:S3–7. 10.1016/j.metabol.2017.01.012 28159329 PMC6510241

[53] Elias WJ Lipsman N Ondo WG Ghanouni P Kim YG Lee W. A randomized trial of focused ultrasound thalamotomy for essential tremor. N Engl J Med. (2016) 375:730–9. 10.1056/NEJMc1612210 27557301

[54] Elias WJ Huss D Voss T Loomba J Khaled M Zadicario E. A pilot study of focused ultrasound thalamotomy for essential tremor. N Engl J Med. (2013) 369:640–8. 10.1056/NEJMoa1300962 23944301

[55] Zhu L Huang Y Lam D Gach HM Zoberi I Hallahan DE. Targetability of cervical cancer by magnetic resonance-guided high-intensity focused ultrasound (MRgHIFU)-mediated hyperthermia (HT) for patients receiving radiation therapy. Int J Hyperthermia. (2021) 38:498–510. 10.1080/02656736.2021.1895330 33757406

[56] Singh MP Sethuraman SN Miller C Malayer J Ranjan A. Boiling histotripsy and in-situ CD40 stimulation improve the checkpoint blockade therapy of poorly immunogenic tumors. Theranostics. (2021) 11:540–54. 10.7150/thno.49517 33391491 PMC7738858

[57] Sethuraman SN Singh MP Patil G Li S Fiering S Hoopes PJ. Novel calreticulin-nanoparticle in combination with focused ultrasound induces immunogenic cell death in melanoma to enhance antitumor immunity. Theranostics. (2020) 10:3397–412. 10.7150/thno.42243 32206098 PMC7069083

[58] Mead BP Mastorakos P Suk JS Klibanov AL Hanes J Price RJ. Targeted gene transfer to the brain via the delivery of brain-penetrating DNA nanoparticles with focused ultrasound. J Control Release. (2016) 223:109–17. 10.1016/j.jconrel.2015.12.034 26732553 PMC4739627

[59] Wei HJ Upadhyayula PS Pouliopoulos AN Englander ZK Zhang X Jan CI. Focused ultrasound-mediated blood-brain barrier opening increases delivery and efficacy of etoposide for glioblastoma treatment. Int J Radiat Oncol Biol Phys. (2021) 110:539–50. 10.1016/j.ijrobp.2020.12.019 33346092 PMC8553628

[60] McMahon D Bendayan R Hynynen K. Acute effects of focused ultrasound-induced increases in blood-brain barrier permeability on rat microvascular transcriptome. Sci Rep. (2017) 7:45657. 10.1038/srep45657 28374753 PMC5379491

[61] Kovacs ZI Kim S Jikaria N Qureshi F Milo B Lewis BK. Disrupting the blood-brain barrier by focused ultrasound induces sterile inflammation. Proc Natl Acad Sci U S A. (2017) 114:E75–84. 10.1073/pnas.1614777114 27994152 PMC5224365

[62] Wang S Frenkel V Zderic V. Optimization of pulsed focused ultrasound exposures for hyperthermia applications. J Acoust Soc Am. (2011) 130:599–609. 10.1121/1.3598464 21786925

[63] O'Neill BE Vo H Angstadt M Li KP Quinn T Frenkel V. Pulsed high intensity focused ultrasound mediated nanoparticle delivery: mechanisms and efficacy in murine muscle. Ultrasound Med Biol. (2009) 35:416–24. 10.1016/j.ultrasmedbio.2008.09.021 19081668 PMC2668521

[64] Frenkel V Etherington A Greene M Quijano J Xie J Hunter F. Delivery of liposomal doxorubicin (Doxil) in a breast cancer tumor model: investigation of potential enhancement by pulsed-high intensity focused ultrasound exposure. Acad Radiol. (2006) 13:469–79. 10.1016/j.acra.2005.08.024 16554227

[65] Vykhodtseva NI Hynynen K Damianou C. Histologic effects of high intensity pulsed ultrasound exposure with subharmonic emission in rabbit brain *in vivo*. Ultrasound Med Biol. (1995) 21:969–79. 10.1016/0301-5629(95)00038-S 7491751

[66] Hynynen K McDannold N Vykhodtseva N Jolesz FA. Noninvasive MR imaging-guided focal opening of the blood-brain barrier in rabbits. Radiology. (2001) 220:640–6. 10.1148/radiol.2202001804 11526261

[67] Burgess A Shah K Hough O Hynynen K. Focused ultrasound-mediated drug delivery through the blood-brain barrier. Expert Rev Neurother. (2015) 15:477–91. 10.1586/14737175.2015.1028369 25936845 PMC4702264

[68] Fishman PS Frenkel V. Treatment of movement disorders with focused ultrasound. J Central Nerv Syst Dis. (2017) 9:117957351770567. 10.1177/1179573517705670 28615985 PMC5462491

[69] Meng Y Jones RM Davidson B Huang Y Pople CB Surendrakumar S.Technical principles and clinical workflow of transcranial MR-guided focused ultrasound. Stereotact Funct Neurosurg. (2020). 10.1159/000512111. [Epub ahead of print]. 33302282

[70] Huang Y Alkins R Schwartz ML Hynynen K. Opening the blood-brain barrier with MR imaging-guided focused ultrasound: preclinical testing on a trans-human skull porcine model. Radiology. (2017) 282:123–30. 10.1148/radiol.2016152154 27420647 PMC5179309

[71] Rivens I Shaw A Civale J Morris H. Treatment monitoring and thermometry for therapeutic focused ultrasound. Int J Hyperthermia. (2007) 23:121–39. 10.1080/02656730701207842 17578337

[72] Treat LH McDannold N Vykhodtseva N Zhang Y Tam K Hynynen K. Targeted delivery of doxorubicin to the rat brain at therapeutic levels using MRI-guided focused ultrasound. Int J Cancer. (2007) 121:901–7. 10.1002/ijc.22732 17437269

[73] Mainprize T Lipsman N Huang Y Meng Y Bethune A Ironside S. Blood-brain barrier opening in primary brain tumors with non-invasive MR-guided focused ultrasound: a clinical safety and feasibility study. Sci Rep. (2019) 9:321. 10.1038/s41598-018-36340-0 30674905 PMC6344541

[74] Lipsman N Meng Y Bethune AJ Huang Y Lam B Masellis M. Blood-brain barrier opening in Alzheimer's disease using MR-guided focused ultrasound. Nat Commun. (2018) 9:2336. 10.1038/s41467-018-04529-6 30046032 PMC6060168

[75] Abrahao A Meng Y Llinas M Huang Y Hamani C Mainprize T. First-in-human trial of blood–brain barrier opening in amyotrophic lateral sclerosis using MR-guided focused ultrasound. Nat Commun. (2019) 10:4373. 10.1038/s41467-019-12426-9 31558719 PMC6763482

[76] Jordao JF Thevenot E Markham-Coultes K Scarcelli T Weng YQ Xhima K. Amyloid-beta plaque reduction, endogenous antibody delivery and glial activation by brain-targeted, transcranial focused ultrasound. Exp Neurol. (2013) 248:16–29. 10.1016/j.expneurol.2013.05.008 23707300 PMC4000699

[77] Burgess A Ayala-Grosso CA Ganguly M Jordão JF Aubert I Hynynen K. Targeted delivery of neural stem cells to the brain using MRI-guided focused ultrasound to disrupt the blood-brain barrier. PLoS ONE. (2011) 6:e27877. 10.1371/journal.pone.0027877 22114718 PMC3218061

[78] Lee J Chang WS Shin J Seo Y Kong C Song BW. Non-invasively enhanced intracranial transplantation of mesenchymal stem cells using focused ultrasound mediated by overexpression of cell-adhesion molecules. Stem Cell Res. (2020) 43:101726. 10.1016/j.scr.2020.101726 32028085

[79] Alkins R Burgess A Ganguly M Francia G Kerbel R Wels WS. Focused ultrasound delivers targeted immune cells to metastatic brain tumors. Cancer Res. (2013) 73:1892–99. 10.1158/0008-5472.CAN-12-2609 23302230 PMC3607446

[80] Alkins R Burgess A Kerbel R Wels WS Hynynen K. Early treatment of HER2-amplified brain tumors with targeted NK-92 cells and focused ultrasound improves survival. Neuro Oncol. (2016) 18:974–81. 10.1093/neuonc/nov318 26819443 PMC4896543

[81] Shen WB Anastasiadis P Nguyen B Yarnell D Yarowsky PJ Frenkel V. Magnetic enhancement of stem cell-targeted delivery into the brain following MR-guided focused ultrasound for opening the blood-brain barrier. Cell Transplant. (2017) 26:1235–46. 10.1177/0963689717715824 28933214 PMC5657739

[82] Liu HL Hua MY Yang HW Huang CY Chu PC Wu JS. Magnetic resonance monitoring of focused ultrasound/magnetic nanoparticle targeting delivery of therapeutic agents to the brain. Proc Natl Acad Sci U S A. (2010) 107:15205–10. 10.1073/pnas.1003388107 20696897 PMC2930577

[83] Cui H Zhu Q Xie Q Liu Z Gao Y He Y. Low intensity ultrasound targeted microbubble destruction assists MSCs delivery and improves neural function in brain ischaemic rats. J Drug Target. (2020) 28:320–9. 10.1080/1061186X.2019.1656724 31429596

[84] Kalkowski L Malysz-Cymborska I Golubczyk D Janowski M Holak P Milewska K. MRI-guided intracerebral convection-enhanced injection of gliotoxins to induce focal demyelination in swine. PLoS ONE. (2018) 13:e0204650. 10.1371/journal.pone.0204650 30273376 PMC6166947

[85] Ruster B Gottig S Ludwig RJ Bistrian R Muller S Seifried E. Mesenchymal stem cells display coordinated rolling and adhesion behavior on endothelial cells. Blood. (2006) 108:3938–44. 10.1182/blood-2006-05-025098 16896152

[86] Prowse AB Chong F Gray PP Munro TP. Stem cell integrins: implications for *ex-vivo* culture and cellular therapies. Stem Cell Res. (2011) 6:1–12. 10.1016/j.scr.2010.09.005 21075697

[87] Ziadloo A Burks SR Gold EM Lewis BK Chaudhry A Merino MJ. Enhanced homing permeability and retention of bone marrow stromal cells by noninvasive pulsed focused ultrasound. Stem Cells. (2012) 30:1216–27. 10.1002/stem.1099 22593018 PMC3356926

[88] Burks SR Ziadloo A Hancock HA Chaudhry A Dean DD Lewis BK. Investigation of cellular and molecular responses to pulsed focused ultrasound in a mouse model. PLoS ONE. (2011) 6:e24730. 10.1371/journal.pone.0024730 21931834 PMC3172304

[89] Burks SR Ziadloo A Kim SJ Nguyen BA Frank JA. Noninvasive pulsed focused ultrasound allows spatiotemporal control of targeted homing for multiple stem cell types in murine skeletal muscle and the magnitude of cell homing can be increased through repeated applications. Stem Cells. (2013) 31:2551–60. 10.1002/stem.1495 23922277 PMC3834159

[90] Burks SR Nguyen BA Tebebi PA Kim SJ Bresler MN Ziadloo A. Pulsed focused ultrasound pretreatment improves mesenchymal stromal cell efficacy in preventing and rescuing established acute kidney injury in mice. Stem Cells. (2015) 33:1241–53. 10.1002/stem.1965 25640064 PMC4376574

[91] Tebebi PA Kim SJ Williams RA Milo B Frenkel V Burks SR. Improving the therapeutic efficacy of mesenchymal stromal cells to restore perfusion in critical limb ischemia through pulsed focused ultrasound. Sci Rep. (2017) 7:41550. 10.1038/srep41550 28169278 PMC5294408

[92] Jang KW Tu TW Rosenblatt RB Burks SR Frank JA. MR-guided pulsed focused ultrasound improves mesenchymal stromal cell homing to the myocardium. J Cell Mol Med. (2020) 24:13278–88. 10.1111/jcmm.15944 33067927 PMC7701528

[93] Burks SR Lorsung RM Nagle ME Tu TW Frank JA. Focused ultrasound activates voltage-gated calcium channels through depolarizing TRPC1 sodium currents in kidney and skeletal muscle. Theranostics. (2019) 9:5517–31. 10.7150/thno.33876 31534500 PMC6735402

[94] Burks SR Nguyen BA Bresler MN Nagle ME Kim SJ Frank JA. Anti-inflammatory drugs suppress ultrasound-mediated mesenchymal stromal cell tropism to kidneys. Sci Rep. (2017) 7:8607. 10.1038/s41598-017-08887-x 28819122 PMC5561076

[95] Hancock HA Smith LH Cuesta J Durrani AK Angstadt M Palmeri ML. Investigations into pulsed high-intensity focused ultrasound-enhanced delivery: preliminary evidence for a novel mechanism. Ultrasound Med Biol. (2009) 35:1722–36. 10.1016/j.ultrasmedbio.2009.04.020 19616368 PMC2752481

[96] Ziadloo A Xie J Frenkel V. Pulsed focused ultrasound exposures enhance locally administered gene therapy in a murine solid tumor model. J Acoust Soc Am. (2013) 133:1827–34. 10.1121/1.4789390 23464051 PMC3606298

[97] Hersh DS Anastasiadis P Mohammadabadi A Nguyen BA Guo S Winkles JA. MR-guided transcranial focused ultrasound safely enhances interstitial dispersion of large polymeric nanoparticles in the living brain. PLoS ONE. (2018) 13:e0192240. 10.1371/journal.pone.0192240 29415084 PMC5802894

[98] Tebebi PA Burks SR Kim SJ Williams RA Nguyen BA Venkatesh P. Cyclooxygenase-2 or tumor necrosis factor-alpha inhibitors attenuate the mechanotransductive effects of pulsed focused ultrasound to suppress mesenchymal stromal cell homing to healthy and dystrophic muscle. Stem Cells. (2015) 33:1173–86. 10.1002/stem.1927 25534849 PMC4376568

[99] Xhima K Nabbouh F Hynynen K Aubert I Tandon A. Noninvasive delivery of an alpha-synuclein gene silencing vector with magnetic resonance-guided focused ultrasound. Mov Disord. (2018) 33:1567–79. 10.1002/mds.101 30264465 PMC6282171

[100] Provenzale JM Silva GA. Uses of nanoparticles for central nervous system imaging and therapy. AJNR Am J Neuroradiol. (2009) 30:1293–301. 10.3174/ajnr.A1590 19617446 PMC7051551

[101] Zheng Y Huang J Zhu T Li R Wang Z Ma F. Stem cell tracking technologies for neurological regenerative medicine purposes. Stem Cells Int. (2017) 2017:9. 10.1155/2017/2934149 29138636 PMC5613625

[102] Gu E Chen WY Gu J Burridge P Wu JC. Molecular imaging of stem cells: tracking survival, biodistribution, tumorigenicity, and immunogenicity. Theranostics. (2012) 2:335–45. 10.7150/thno.3666 22509197 PMC3326720

[103] Zhu J Zhou L XingWu F. Tracking neural stem cells in patients with brain trauma. N Engl J Med. (2006) 355:2376–8. 10.1056/NEJMc055304 17135597

[104] Bulte JWM Daldrup-Link HE. Clinical tracking of cell transfer and cell transplantation: trials and tribulations. Radiology. (2018) 289:604–15. 10.1148/radiol.2018180449 30299232 PMC6276076

[105] Gorelik M Orukari I Wang J Galpoththawela S Kim H Levy M. Use of MR cell tracking to evaluate targeting of glial precursor cells to inflammatory tissue by exploiting the very late antigen-4 docking receptor. Radiology. (2012) 265:175–85. 10.1148/radiol.12112212 22923719 PMC3447172

[106] Richard J-P Hussain U Gross S Taga A Kouser M Almad A. Perfluorocarbon labeling of human glial-restricted progenitors for 19F magnetic resonance imaging. Stem Cells Transl Med. (2019) 8:355–65. 10.1002/sctm.18-0094 30618148 PMC6431733

[107] Cheng SH Yu D Tsai HM Morshed RA Kanojia D Lo LW. Dynamic *in vivo* SPECT imaging of neural stem cells functionalized with radiolabeled nanoparticles for tracking of glioblastoma. J Nucl Med. (2016) 57:279–84. 10.2967/jnumed.115.163006 26564318 PMC5831675

[108] Bulte JW Walczak P Janowski M Krishnan KM Arami H Halkola A. Quantitative hot spot imaging of transplanted stem cells using superparamagnetic tracers and magnetic particle imaging (MPI). Tomography. (2015) 1:91–7. 10.18383/j.tom.2015.00172 26740972 PMC4699415

[109] Kennis BA Michel KA Brugmann WB Laureano A Tao R-H Somanchi SS. Monitoring of intracerebellarly-administered natural killer cells with fluorine-19 MRI. J Neuro Oncol. (2019) 142:395–407. 10.1007/s11060-019-03091-5 30788681 PMC11492566

[110] Bose RJC Mattrey RF. Accomplishments and challenges in stem cell imaging *in vivo*. Drug Discovery Today. (2019) 24:492–504. 10.1016/j.drudis.2018.10.007 30342245

[111] Tannous BA Grimm J Perry KF Chen JW Weissleder R Breakefield XO. Metabolic biotinylation of cell surface receptors for in vivo imaging. Nat Methods. (2006) 3:391–6. 10.1038/nmeth875 16628210

[112] Bindslev L Haack-Sørensen M Bisgaard K Kragh L Mortensen S Hesse B. Labelling of human mesenchymal stem cells with indium-111 for SPECT imaging: effect on cell proliferation and differentiation. Eur J Nucl Med Mol Imaging. (2006) 33:1171–7. 10.1007/s00259-006-0093-7 16763813

[113] Gildehaus FJ Haasters F Drosse I Wagner E Zach C Mutschler W. Impact of Indium-111 oxine labelling on viability of human mesenchymal stem cells *in vitro*, and 3D cell-tracking using SPECT/CT *in vivo*. Mol Imaging Biol. (2011) 13:1204–14. 10.1007/s11307-010-0439-1 21080231

[114] Varma NR Janic B Iskander AS Shankar A Bhuiyan MP Soltanian-Zadeh H. Endothelial progenitor cells (EPCs) as gene carrier system for rat model of human glioma. PLoS ONE. (2012) 7:e30310. 10.1371/journal.pone.0030310 22276177 PMC3262815

[115] Wolfs E Struys T Notelaers T Roberts SJ Sohni A Bormans G. 18F-FDG labeling of mesenchymal stem cells and multipotent adult progenitor cells for PET imaging: effects on ultrastructure and differentiation capacity. J Nucl Med. (2013) 54:447–54. 10.2967/jnumed.112.108316 23353687

[116] Holvoet B De Waele L Quattrocelli M Gheysens O Sampaolesi M Verfaillie CM. Increased understanding of stem cell behavior in neurodegenerative and neuromuscular disorders by use of noninvasive cell imaging. Stem Cells Int. (2016) 2016:6235687. 10.1155/2016/6235687 26997958 PMC4779824

[117] Ludewig P Gdaniec N Sedlacik J Forkert ND Szwargulski P Graeser M. Magnetic particle imaging for real-time perfusion imaging in acute stroke. ACS Nano. (2017) 11:10480–8. 10.1021/acsnano.7b05784 28976180

[118] Nejadnik H Pandit P Lenkov O Lahiji AP Yerneni K Daldrup-Link HE. Ferumoxytol can be used for quantitative magnetic particle imaging of transplanted stem cells. Mol Imaging Biol. (2019) 21:465–72. 10.1007/s11307-018-1276-x30194566

[119] Graeser MFT Szwargulski P Werner F Gdaniec N Boberg M Griese F. Human-sized magnetic particle imaging for brain applications. Nat Commun. (2019) 10:1936. 10.1038/s41467-019-09704-x 31028253 PMC6486595

[120] Klauer P Vogel P Rückert MA Kullmann WH Jakob PM Behr VC. Bimodal TWMPI-MRI hybrid scanner—coil setup and electronics. IEEE Transac Magnet. (2015) 51:1–4. 10.1109/TMAG.2014.2324180

[121] Dorsey ER Sherer T Okun MS Bloem BR. The emerging evidence of the Parkinson pandemic. J Parkinsons Dis. (2018) 8:S3–8. 10.3233/JPD-181474 30584159 PMC6311367

[122] Liu Z Cheung HH. Stemcell-based therapies for Parkinson disease. Int J Mol Sci. (2020) 21. 10.3390/ijms21218060 PMC7663462 33137927

[123] Thomsen GM Avalos P Ma AA Alkaslasi M Cho N Wyss L. Transplantation of neural progenitor cells expressing glial cell line-derived neurotrophic factor into the motor cortex as a strategy to treat amyotrophic lateral sclerosis. Stem Cells. (2018) 36:1122–31. 10.1002/stem.2825 29656478

[124] Parmar M Grealish S Henchcliffe C. The future of stem cell therapies for Parkinson disease. Nat Rev Neurosci. (2020) 21:103–15. 10.1038/s41583-019-0257-7 31907406

[125] Downs ME Buch A Sierra C Karakatsani ME Teichert T Chen S. Long-term safety of repeated blood-brain barrier opening via focused ultrasound with microbubbles in non-human primates performing a cognitive task. PLoS ONE. (2015) 10:e0125911. 10.1371/journal.pone.0125911 25945493 PMC4422704

[126] van Linde ME Brahm CG de Witt Hamer PC Reijneveld JC Bruynzeel AME Vandertop WP. Treatment outcome of patients with recurrent glioblastoma multiforme: a retrospective multicenter analysis. J Neurooncol. (2017) 135:183–92. 10.1007/s11060-017-2564-z 28730289 PMC5658463

[127] Ballabh P Braun A Nedergaard M. The blood-brain barrier: an overview: structure, regulation, clinical implications. Neurobiol Dis. (2004) 16:1–13. 10.1016/j.nbd.2003.12.016 15207256

[128] Bago JR Alfonso-Pecchio A Okolie O Dumitru R Rinkenbaugh A Baldwin AS. Induced neural stem cells are tumour-homing and inhibit progression of glioblastoma. Nat Commun. (2016) 7:10593. 10.1038/ncomms10593 26830441 PMC4740908

[129] Sharif S Ghahremani MH Soleimani M. Delivery of exogenous miR-124 to glioblastoma multiform cells by wharton's jelly mesenchymal stem cells decreases cell proliferation and migration, confers chemosensitivity. Stem Cell Rev Rep. (2018) 14:236–46. 10.1007/s12015-017-9788-3 29185191

[130] Portnow J Synold TW Badie B Tirughana R Lacey SF D'Apuzzo M. NSC-based anticancer gene therapy: a first-in-human study in recurrent high-grade glioma patients. Clin Cancer Res. (2017) 23:2951–60. 10.1158/1078-0432.CCR-16-1518 27979915 PMC8843778

[131] Park SH Kim MJ Jung HH Chang WS Choi HS Rachmilevitch I. Safety and feasibility of multiple blood-brain barrier disruptions for the treatment of glioblastoma in patients undergoing standard adjuvant chemotherapy. J Neurosurg. (2020) 134:1–9. 10.3171/2019.10.JNS192206 31899873

[132] Xiao J Yang R Biswas S Zhu Y Qin X Zhang M. NSC-based regenerative treatment of multiple sclerosis. Mol Neurobiol. (2018) 55:3152–71. 10.1007/s12035-017-0566-7 28466274 PMC5668198

[133] Yousefi F Lavi Arab F Saeidi K Amiri H Mahmoudi M. Various strategies to improve efficacy of stem cell transplantation in multiple sclerosis: focus on mesenchymal stem cells and neuroprotection. J Neuroimmunol. (2019) 328:20–34. 10.1016/j.jneuroim.2018.11.015 30557687

[134] Kim MJ Lim JY Park SA Park SI Kim WS Ryu CH. Effective combination of methylprednisolone and interferon beta-secreting mesenchymal stem cells in a model of multiple sclerosis. J Neuroimmunol. (2018) 314:81–8. 10.1016/j.jneuroim.2017.11.010 29224961

[135] Connick P Kolappan M Crawley C Webber DJ Patani R Michell AW. Autologous mesenchymal stem cells for the treatment of secondary progressive multiple sclerosis: an open-label phase 2a proof-of-concept study. Lancet Neurol. (2012) 11:150–6. 10.1016/S1474-4422(11)70305-2 22236384 PMC3279697

[136] Riordan NH Morales I Fernandez G Allen N Fearnot NE Leckrone ME. Clinical feasibility of umbilical cord tissue-derived mesenchymal stem cells in the treatment of multiple sclerosis. J Transl Med. (2018) 16:57. 10.1186/s12967-018-1433-7 29523171 PMC5845260

[137] Dimitrios Karussis M Karageorgiou C Vaknin-Dembinsky A. Safety and Immunological effects of mesenchymal stem cell transplantation in patients with multiple sclerosis and amyotrophic lateral sclerosis. Arch Neurol. (2010) 67:1187–94. 10.1001/archneurol.2010.248 20937945 PMC3036569

[138] Leker RR Shohami E. Cerebral ischemia and trauma—different etiologies yet similar mechanisms: neuroprotective opportunities. Brain Res Rev. (2002) 39:55–73. 10.1016/S0165-0173(02)00157-1 12086708

[139] Steinberg GK Kondziolka D Wechsler LR Lunsford LD Kim AS Johnson JN. Two-year safety and clinical outcomes in chronic ischemic stroke patients after implantation of modified bone marrow-derived mesenchymal stem cells (SB623): a phase 1/2a study. J Neurosurg. (2018) 131:1–11. 10.3171/2018.5.JNS173147 30497166

[140] Nikolic B Faintuch S Goldberg SN Kuo MD Cardella JF. Stem cell therapy: a primer for interventionalists and imagers. J Vasc Interv Radiol. (2009) 20:999–1012. 10.1016/j.jvir.2009.04.075 19647179

[141] Pendharkar AV Chua JY Andres RH Wang N Gaeta X Wang H. Biodistribution of NSCs after intravascular therapy for hypoxic-ischemia. Stroke. (2010) 41:2064–70. 10.1161/STROKEAHA.109.575993 20616329 PMC4831577

